# ER stress promotes mitochondrial DNA mediated type-1 interferon response in beta-cells and interleukin-8 driven neutrophil chemotaxis

**DOI:** 10.3389/fendo.2022.991632

**Published:** 2022-09-12

**Authors:** Saurabh Vig, Joost M. Lambooij, Mette C. Dekkers, Frank Otto, Françoise Carlotti, Bruno Guigas, Arnaud Zaldumbide

**Affiliations:** ^1^ Department of Cell and Chemical Biology Leiden University Medical Center, Leiden, Netherlands; ^2^ Department of Parasitology, Leiden University Medical Center, Leiden, Netherlands; ^3^ Department of Internal Medicine, Leiden University Medical Center, Leiden, Netherlands

**Keywords:** type 1 diabetes, ER stress, mitochondria, innate immunity, neutrophils

## Abstract

Beta-cell destruction in type 1 diabetes (T1D) results from the combined effect of inflammation and recurrent autoimmunity. Accumulating evidence suggests the engagement of cellular stress during the initial stage of the disease, preceding destruction and triggering immune cell infiltration. While the role of the endoplasmic reticulum (ER) in this process has been largely described, the participation of the other cellular organelles, particularly the mitochondria which are central mediator for beta-cell survival and function, remains poorly investigated. Here, we have explored the contribution of ER stress, in activating type-I interferon signaling and innate immune cell recruitment. Using human beta-cell line EndoC-βH1 exposed to thapsigargin, we demonstrate that induction of cellular stress correlates with mitochondria dysfunction and a significant accumulation of cytosolic mitochondrial DNA (mtDNA) that triggers neutrophils migration by an IL8-dependent mechanism. These results provide a novel mechanistic insight on how ER stress can cause insulitis and may ultimately facilitate the identification of potential targets to protect beta-cells against immune infiltration.

## Introduction

The recent comparison of targeted tissues in systemic lupus erythematosus, multiple sclerosis rheumatoid arthritis and in type 1 diabetes (T1D) has positioned the type-I interferon (IFN-I) signature as a common feature of autoimmune diseases (1).

Classically, the induction of the IFN-I response to pathogens is a complex signaling pathway initiated by Pattern Recognition Receptors (PRRS) located at the cell surface (Toll-like receptor (TLR) 1, 2, 4, 5, 6, 10), in endosomes (TLR3, TLR7, TLR8, TLR9, 11, 12, 13) or within the cytosol (retinoic acid-inducible gene I (RIGI), [melanoma differentiation-associated protein 5 (MDA5), cyclic GMP–AMP synthase (cGAS)]. Once triggered, a cascade of events involving adapter proteins (e.g. MYD88 or TRIF), and kinases leads to NFкB activation and transcriptional expression of both inflammatory cytokines and IFN-I associated genes (2, 3).

In T1D, activation of the innate immune response has been detected prior to seroconversion in young children and consequently associated with progression from the prediabetic phase to disease development and recruitment of innate immune cells into the pancreas, notably neutrophils (4–7). While the molecular mechanisms triggering T1D remain unclear, the nPOD virus group recently added further evidence for the implication of enterovirus infection in the IFN-I response, showing direct correlations between the presence of enterovirus proteins and expression of IFN-I markers, loss of beta cell function and the number of infiltrating immune cells in the pancreas (8–10). However, the associations between mitochondria dysfunction and interferonopathy on one hand, and the implication of mtDNA leakage in the development of Systemic Lupus Erythematous (SLE) on the other hand (11–14) may suggest that an endogenous source of pathogen associated molecular patterns (PAMPs) could also activate an antiviral-like signature in target tissues in autoimmune diseases, independently of the presence of a virus or a foreign pathogen.

In response to stress, the close relationship between the ER and the mitochondria indicates that both organelles orchestrate the communication between the beta cells and the immune system compartment (15). All cells, including beta-cells, have an adaptive signaling pathway aimed to restore cellular homeostasis in response to ER stress by i) inhibiting mRNA translation, ii) inducing the ERAD degradation pathway, and iii) promoting transcription of genes encoding for ER chaperones. In addition, induction of ER stress initiates Ca^2+^ release from the ER leading to an increased mitochondrial Ca^2+^ concentration and the induction of apoptosis *via* release of cytochrome C (16, 17). Under these conditions, the increased permeability of the mitochondria may also facilitate the release of mitochondrial genetic material into the cytosol (15, 18, 19).

In the present study we evaluated the impact of ER stress on mitochondrial function in human beta-cell line EndoC-βH1 and demonstrate that ER stress triggers the release of mtDNA into the cytosol leading to the activation of a IFN-I response, subsequently promoting neutrophil migration to the stressed cells. Our data provides novel insight into the role of beta-cells in T1D pathogenesis, suggesting that they may not only contribute to their own destruction but could actually be the sparks initiating the development of autoimmunity.

## Materials and methods

### Cells and reagents

EndoC-βH1 cells, obtained from Dr. Raphael Scharfmann (Paris Descartes University, France) (20), were maintained as described previously (21). ER stress was induced 100nM Thapsigargin (TG; Adipogen Life sciences, Fuellinsdorf, Switzerland) for 24h.

### Transfections and transductions

For stimulation of nucleic acid-sensing pathway in EndoC-βH1, we transfected the cells with either polyinosinic-polycytidylic acid (poly(I:C); 5μg/ml) to mimic double stranded RNA (dsRNA) or fluorescein amidite (FAM)-labelled double stranded DNA (dsDNA) (derived from the HSV-1 genome; 1μg/ml) (22) to mimic exogenous DNA using lipofectamine 2000 (Invitrogen, Germany) as per manufacturer’s instructions. Cells were harvested 24h post-transfection and transfection efficiency was measured by either flow cytometry or gene expression determined by RT-qPCR. PolyI:C transfection efficiency was determined after staining with an anti-dsRNA antibody J2 (dilution 1:100) (Jena Bioscience) and Alexa fluro-647 labeled GAM secondary antibody. dsDNA transfection efficiency was determined using FAM fluorescence of the transfected dsDNA.

MDA5 downregulation was achieved by siRNA-smartpool (M-013041-00-0005; Horizon Discovery, Cambridge, UK) transfection using DharmaFECT 1 Transfection Reagent (Horizon Discovery) as per manufacturer’s instructions for 48h, followed by exposure to TG (0.1µM; 24h). The knockdown was analyzed RT-qPCR using gene specific primers.

cGAS knockdown EndoC-βH1 line was generated by lentiviral transduction of shRNA against cGAS (Sigma; TRCN0000146282) or negative control (Sigma; SHC002). Lentiviruses were produced as described before (23). EndoC-βH1 cells were cultured a day before transduction. Cells were incubated with virus supernatant and polybrene (8μg/ml) for 24h. After incubation, the virus medium was changed with fresh medium and the cells were incubated for 3 days, followed by puromycin (3μg/ml) selection. cGAS knockdown was analyzed RT-qPCR.

### Intracellular dsRNA staining

The cells were collected after treatment and fixed with 4% Paraformaldehyde solution containing 0.1% Saponin for 20 min at 4°C; followed by three washings with wash buffer (phosphate-buffered saline (PBS), 0.1% Saponin), blocking with PBS, 0.2% BSA, 0.1% Saponin; 10min at 4°C and incubation with anti-dsRNA monoclonal J2 antibody (1:500; diluted in blocking buffer; 30 min at 4°C)(Sigma-Aldrich, Amsterdam, Netherlands). The cells were washed three times with wash buffer and incubated with Alexa-fluro-647 labeled Goat anti-mouse antibody (1:500; diluted in blocking buffer)(Invitrogen, Karlsruhe, Germany) for 30 min at 4°C; followed by washing with PBS and acquisition on LSRII flow cytometer (BD Biosciences, San Jose, CA, USA). Analysis of flow cytometry data was performed using Flowjo software (BD, Ashland, USA).

### RNA isolation, cDNA synthesis and RT-qPCR

RNA was isolated by using an RNA isolation kit (Bioké, Leiden, Netherlands), according to the manufacturer’s protocol. cDNA was synthesized from 500-1000ng of RNA, using Oligo-dT primer and Superscript II Reverse Transcriptase (Invitrogen); 5ng of cDNA was used per 10μl reaction of Reverse transcription polymerase chain reaction (RT-qPCR), containing 5 pmol of gene-specific primers (see **Table 1**) and iQ SYBR^®^ Green Supermix (Bio-Rad, Veenendaal, Netherlands). Assays were run on CFX Connect Real-Time PCR Detection System (Bio-Rad). Data are presented as 2 ^-ΔΔCT^ (24).

**Table 1:** List of primers.

Gene Symbol	Forward (5'-3')	Reverse (5'-3')
**Housekeeping**		
GAPDH	ACAGTCAGCCGCATCTTCTT	AATGAAGGGGTCATT GATGG
**ER Stress**		
XBP1s	CTGAGTCCGCAGCAGGTG	GAGATGTTCTGGAGGGGTGA
BIP	GACGCTGGAACTATTGCTGG	CTCCCTCTTATCCAGGCCAT
**Mitochondrial Stress**		
HSPA9	TTGGAGCATTTGTGTTGATGA	CCTGTCTCTGCGAGTCATTG
ATF5	GGCTCCCTATGAGGTCCTTG	TCGCTCAGTCATCCAGTCAG
DNAJA3	TGGCGAGTTCTCATCCTCTT	ACTCCTTGTTGACCCCCTTT
**Type-1 Interferon**		
MDA5	GTTGCTCACAGTGGTTCAGG	GCTTGGCAATATTTCTCTTGGT
cGAS	GGGAGCCCTGCTGTAACACTTCTTAT	CCTTTGCATGCTTGGGTACAAGGT
IFNβ	AGGACAGGATGAACTTTGAC	TGATAGACATTAGCCAGGAG
IFIT1	CAGAATAGCCAGATCTCAGAGG	CCAGACTATCCTTGACCTGATG
IFI27	CAGCCTTGTGGCTACTC	GCAATGGCAGACCCAATG
IL8	CTGCGCCAACACAGAAATTA	CTCTGCACCCAGTTTTCCTT
**DNA quantification**		
B2M	TGCTGTCTCCATGTTTGATGTATCT	TCTCTGCTCCCCACCTCTAAGT
tRNALeu(UUR)	CACCCAAGAACAGGGTTTGT	TGGCCATGGGTATGTTGTTA

### Cytosolic mtDNA quantification

The amount of cytosolic mtDNA leakage was determined by quantification of mtDNA in the cytosolic extract (25). Briefly, the cells were collected after stimulation with TG for 24h, resuspended in 1% NP-40 buffer, incubated for 15min on ice and spin down at 13,000 x g for 15min at 4°C. The supernatant (cytosolic extract) was used to isolate cytosolic DNA using DNeasy Blood & Tissue Kit (Qiagen) and the pellet was used to isolate total DNA. Cytosolic mtDNA was quantified in DNA isolated from the cytosolic extract by RT-qPCR using mitochondrial DNA specific gene tRNA leucine 1 (tRNA-LeuUUR) and normalized by nuclear gene (β2 microglobulin) in total DNA (26). Ct values for cytosolic mtDNA was normalized with Ct values of total DNA and relative mtDNA were calculated and plotted.

### Seahorse analyses

Real-time analysis of the oxygen consumption rate (OCR) was measured in EndoC-βH1 cells using a XF-96 Extracellular Flux Analyzer (Seahorse Bioscience). In short, cells were seeded in an XF-96 cell culture plate (Seahorse Bioscience) (30,000 cells/well), grown to confluence and next treated with 0.1uM TG for 24h or left unstimulated. After stimulation, the cells were washed and analyzed in XF culture medium consisting of unbuffered RPMI (Sigma-Aldrich) supplemented with 5% FCS (Serana), 100U/ml penicillin/streptomycin (Sigma-Aldrich) and 2mM L-glutamine (Sigma-Aldrich). Real-time changes in OCR were measured as described by the manufacturer’s protocol in response to 10mM D-glucose (Sigma-Aldrich), 1.5µM oligomycin (Cayman Chemical), 1µM fluoro-carbonyl cyanide phenylhydrazone (FCCP, Sigma-Aldrich) and a combination of 1µM Rotenone and 1µM Antimycin A (Sigma-Aldrich).

### Immune fluorescence staining

To analyze the cytosolic accumulation of dsDNA, the EndoC-βH1 cells were incubated with an anti-dsDNA antibody (ab27156, Abcam, Cambridge, UK). Briefly, the cells were washed with PBS, fixed and permeabilized with a methanol/acetone (70%/30%) solution at -20°C for 10 min, blocked for 1h with 5% skim milk dissolved in PBS at room temperature and incubated with the anti-dsDNA antibody (1:1000; 5% milk in PBS) overnight at 4°C. Next, the cells were washed thrice with PBS and incubated with anti-mouse IgG2a-Alexa fluor-568 antibody (1:1000; 5% skim milk in PBS; Invitrogen) for 1h at room temperature; followed by washings with PBS, mounting with vectashield mounting medium (Vector Laboratories, Newark, USA) and analyzed under Leica SP8 WLL (Leica Microsystems, Amsterdam, Netherlands) confocal microscope with 40x oil immersion objective.

For colocalization of dsDNA with mitochondria, the cells were sequentially co-stained with anti-dsDNA and anti-mitochondria antibody (ab92824; Abcam). First, the cells were stained with primary anti-dsDNA and secondary anti-mouse IgG2a-alexa fluor-568 antibody (Invitrogen) as described above; followed by staining with primary anti-mitochondria antibody (1:1000 in 5% skim milk dissolved in PBS) and secondary antibody anti-mouse-IgG1-Alexa fluor 488. Staining with anti-mitochondria were performed with an incubation time of 1h at room temperature. The cells were analyzed in Leica SP8 WLL (Leica Microsystems) confocal microscope with a 40x oil immersion objective.

### Cytosolic dsDNA quantification in immunocytochemistry

The cytosolic dsDNA quantification was performed as previously described (27) using FIJI software (28). Briefly, the total fluorescence of the cells was measured by tracing the outline of a cell and defined as “X”. Similarly, the fluorescence of the nucleus of the same cell was measured and defined as “Y”. The rate of cytosolic dsDNA was measured and plotted as (X-Y)/Y. The quantification was done on 20-25 cells per condition from different randomly selected images of the same condition.

### Caspase 3/7 activity

The apoptosis on stimulation with chemicals was analyzed by caspase-3/7 activity using the Caspase-Glo^®^ 3/7 Assay (Promega, Leiden, Netherlands), as per the manufacturer’s instructions.

### Measurement of adenine nucleotide levels

Adenine nucleotide concentrations were determined in cell extracts by high-performance liquid chromatography, as described previously (29). Briefly, EndoC-βH1 cells (1 x 10^6^ cells) were treated as described in the figure legend, then medium was removed and cells were scrapped into 6% (v/v) ice-cold HClO_4_. After centrifugation (10 000 x g for 2 minutes at 4°C), the supernatant was collected, neutralized and stored at -80c until subsequent determination of ATP and ADP.

### Neutrophils isolation and transwell migration assay

Neutrophils were isolated from venous blood/buffy coats purchased from Sanquin (The Netherlands) using the Ficoll-Paque density gradient. Briefly, transverse EDTA blood was mixed with PBS (1:1), added to Ficoll-Paque density gradient media (GE Healthcare, IL,USA), and centrifuge at 600 x g for 30min at room temperature with lowest acceleration and brake. The bottom layer of red blood cells (RBCs) and granulocytes were collected, and RBCs were subsequently lysed with sterile MilliQ water for 20sec, washed with PBS and resuspended in RPMI medium (Invitrogen) supplemented with 10% FBS (Invitrogen) and pen/strep (Invitrogen). The neutrophils were counted and subsequently used in a migration assay. The migration assay was performed using 6.5 mm Transwell with 3 μm PVP-free polycarbonate filter membrane (Corning, Amsterdam, Netherlands) in a 24-well micro chemotaxis chamber. Neutrophils were labelled with CMTMR-red (C34552; Invitrogen) dye as per the manufacturer’s instructions. Labelled neutrophils (1x 10^5^/100µl) were added in the upper chamber and incubated with 250μl conditioned medium from stimulated EndoC-βH1 cells in the lower chamber for 2h at 37°C. After incubation, the cells were collected from the lower chamber and counted by flow cytometry. Prior to the acquisition by flow cytometry, an equal number of unlabeled beads (BD biosciences) were added per sample which were used to normalize the events measured.

### Human Islets and secreted cytokine profile

Pancreatic islets were obtained from human organ donor pancreata. Human islets were isolated from organ donors. Islets were only studied if they could not be used for clinical purposes and if research consent had been obtained. According to the national law ethics approval is not required for research on donor tissues that cannot be used for clinical transplantation. The isolations were performed in the GMP-facility of LUMC. For experimental use, human islets were maintained in ultra-low attachment plates (Corning, NY 14831) in low glucose DMEM supplemented with 10% FBS, 100 units/ml Penicillin and 100 μg/ml streptomycin and exposed to 1µM thapsigargin for 5h. After treatment, culture medium was replaced and analyzed for cytokine profile 2 days after using Luminex 9-plex kit (BioRad) according to the manufacturer’s protocol. Islet cells were pelleted and RNA isolated to correct Luminex values. Human IL8 secretion from EndoC-βH1 cells exposed to TG was determined by ELISA MAX™ Deluxe Set Human IL-8 (Biolegend) according to the protocol from the manufacturer.

### Statistics

All graphs and statistical analysis were generated using GraphPad Prism 9.01 (GraphPad Software). Data are presented as the mean ± SEM from at least 3 independent experiments. Two-tailed Student’s t-test was performed for mean comparison and statistical significances estimation of two groups. One-way ANOVA was used for multiple comparisons, with Holm-Sidak’s multiple comparisons for significances estimation. Statistical significance was set at p < 0.05.

## Results

### Beta-cell senses exogenous nucleic acids and activates type-I interferon innate immune response

**Figure 1:**
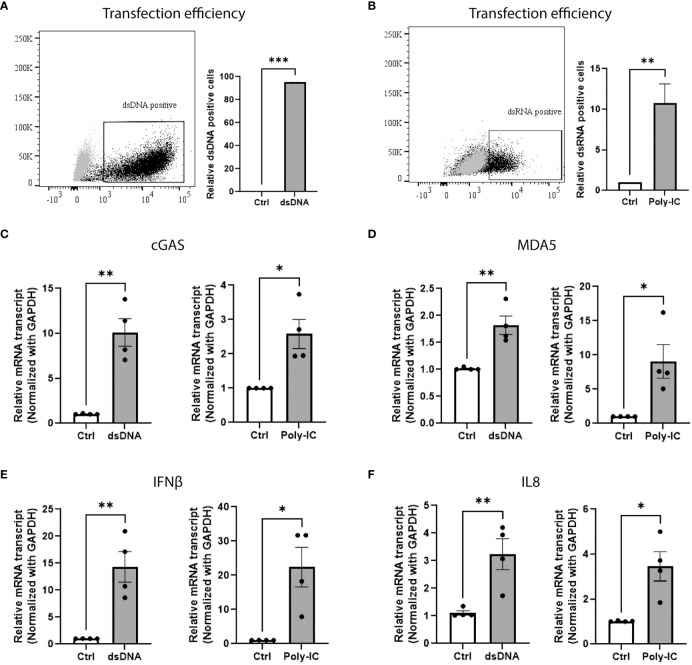
Beta-cell senses exogenous nucleic acids and activates type-I interferon innate immune response. Transfection efficiency of **(A)** FAM-labeled dsDNA and **(B)** Poly-IC in EndoC-βH1 cells. The results are represented by dot plot (left panel) of FACS image and mean ± SEM (right panel) of relative dsDNA and dsRNA positive cells. qPCR analysis of EndoC-βH1 transfected cells showing the relative mRNA expression of cytosolic sensor for **(C)** dsDNA cGAS, **(D)** dsRNA MDA5, **(E)** type 1 interferon gene IFNβ and **(F)** chemokine IL8. Data are presented as mean ± SEM (n = 4) and statistically analyzed by student’s t-test. Significance is indicated by *P < 0.05, **P < 0.01, and ***P < 0.001.

In order to determine whether human beta-cells have functional machinery for sensing foreign nucleic acids, EndoC-βH1 cells were transfected with fam-labelled dsDNA (**Figure 1A**) or Poly-IC (to mimic dsRNA), an experimental approach that has been previously reported and validated (30, 31) (**Figure 1B**). Forty eight hours post transfection, the expression of genes involved in DNA/RNA sensing machinery and in type-I interferon response was assessed by qPCR. As depicted on **Figures 1C, D**, the presence of exogenous DNA and RNA triggered the expression of the cytosolic nucleic acid sensors cGAS and MDA5, respectively, leading to upregulation of IFN-I related genes such as interferon-beta (IFNβ) (**Figure 1E**) and interleukin-8 (IL8) (**Figure 1F**).

### ER stress triggers cytosolic nucleic sensors and IFN-I response in beta-cells

**Figure 2:**
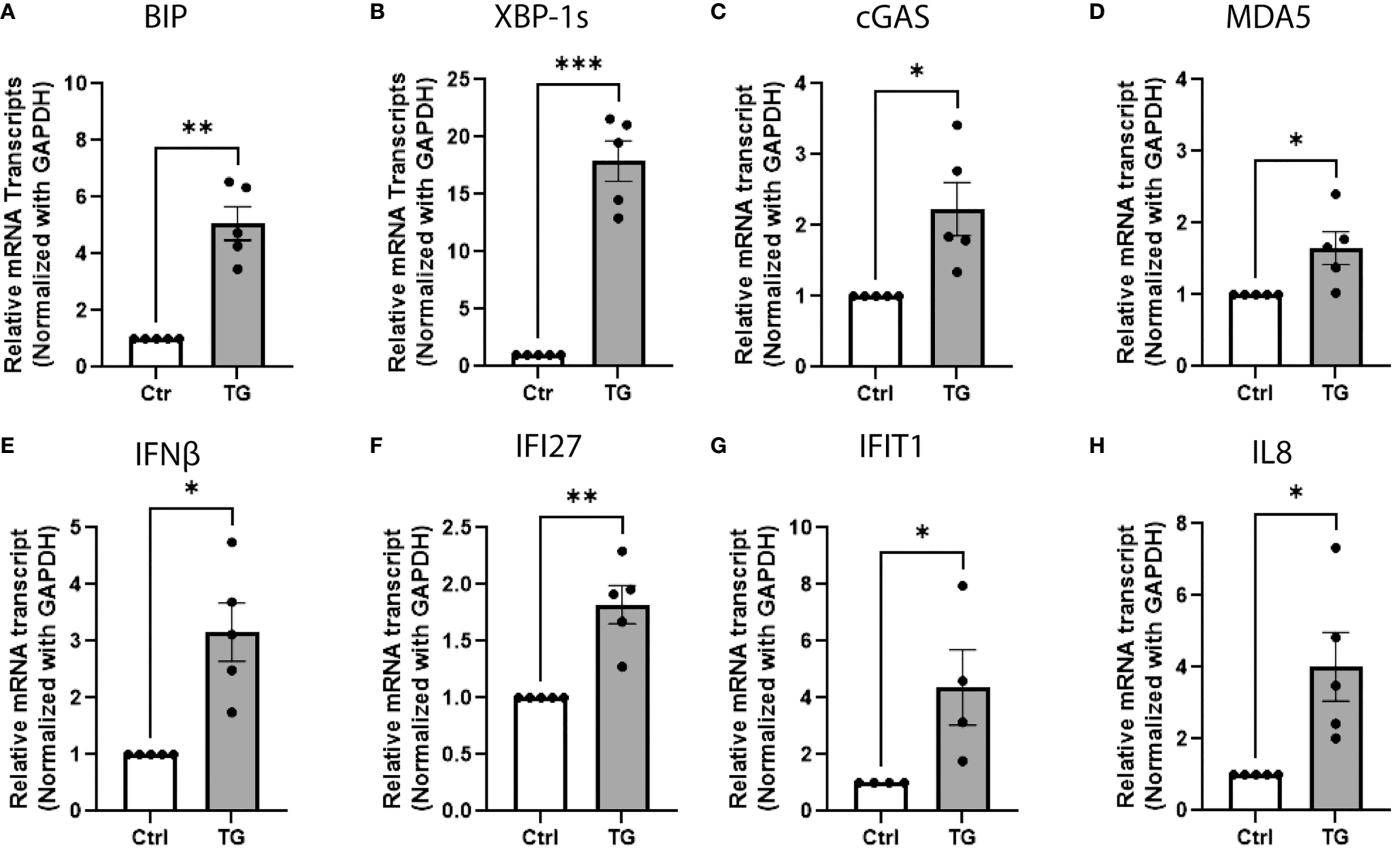
ER stress triggers cytosolic nucleic sensors and IFN-I response in beta-cells. QPCR analysis of EndoC-βH1 treated with thapsigargin (TG; 0.1µM, 24h) showing the relative mRNA expression of **(A, B)** ER stress markers BIP and XBP-1spliced variant, **(C, D)** cytosolic nucleic acid sensors cGAS and MDA5, **(E–G)** type 1 interferon responsive genes IFNβ, IFIT27, IFIT1 and **(H)** chemokine IL8. Data are presented as mean ± SEM (n = 5) and statistically analyzed by student’s t-test. Significance is indicated by *P < 0.05, **P < 0.01, and ***P < 0.001.

Several evidences suggest that cellular stress could initiate an IFN-I response (32–34). To test this hypothesis, EndoC-βH1 cells were treated for 24h with thapsigargin (TG), a Ca^2+^ ATPase inhibitor known as a potent ER stress inducer. In these conditions, TG significantly increased the expression of ER stress markers, such as Binding immunoglobulin protein (BIP) and X-box-binding protein 1spliced variant (XBP-1s) (**Figures 2A, B**), and also led to upregulation of cGAS and MDA5 (**Figures 2C, D** and **Figure S1**), as previously described (35), as well as IFN-I responsive genes, including IFNβ, (Interferon Induced Protein With Tetratricopeptide Repeats 1 (IFIT1), Interferon Alpha Inducible Protein 27 (IFI27), and IL8 (**Figures 2E–H**). Altogether, these results suggest that Ca^2+^-dependent ER stress could trigger the innate immune response secondary to cytosolic accumulation of nucleic acids.

### ER stress promotes cytosolic accumulation of dsDNA, but not dsRNA, in beta-cells

**Figure 3:**
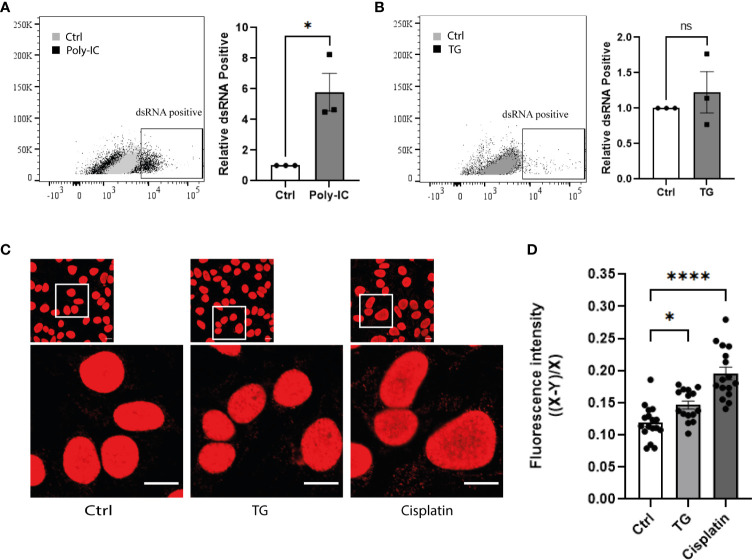
ER stress promotes cytosolic accumulation of dsDNA, but not dsRNA, in beta-cells. Flow cytometry analysis of dsRNA accumulation in EndoC-βH1 cells **(A)** after poly-IC transfection and **(B)** exposure to TG (0.1µM; 24h). Data are represented by dot plot (left panel) of FACS image and mean ± SEM (n=3) of relative dsRNA positive cells (right panel). **(C)** Confocal imaging of EndoC-βH1 cells exposed to TG (0.1µM; 24h) or Cisplatin (1µM; 24h), and stained with anti-dsDNA antibody. One represented image out of three independent experiment is shown. Scale =10µm. **(D)** Quantification of cytosolic dsDNA expression (20-25 cells per condition). Data are presented as mean ± SEM and statistically analyzed by student’s t-test. Significance is indicated by * P < 0.05, ****P < 0.0001. and ns, not significant.

To investigate whether ER stress is associated with elevated levels of cytosolic nucleic acids, dsDNA and dsRNA specific antibodies were used to stain EndoC-βH1 cells after TG exposure. While poly I:C transfection, used as positive control, led to strong accumulation of dsRNA in the cytosol (**Figure 3A**), TG treatment did not affect cytosolic dsRNA (**Figure 3B**). In addition, siRNA-mediated knock down of MDA5 did not affect IFNβ gene expression induced by TG, ruling out dsRNA as potential activator of the type I interferon response (**Figure S2**). In contrast, an immunostaining performed with a dsDNA specific antibody revealed that induction of ER stress was accompanied by an increased dsDNA signal outside nuclei (**Figures 3C, D**), suggesting leakage of genomic material either from the nucleus or the mitochondria. The decreased TG induced IFNβ gene expression observed after cGAS inhibition by shRNA illustrate the role of dsDNA as activator of the type I interferon response (**Figure S2**).

### ER stress induces mitochondrial damage leading to mtDNA leakage to the cytosol

**Figure 4:**
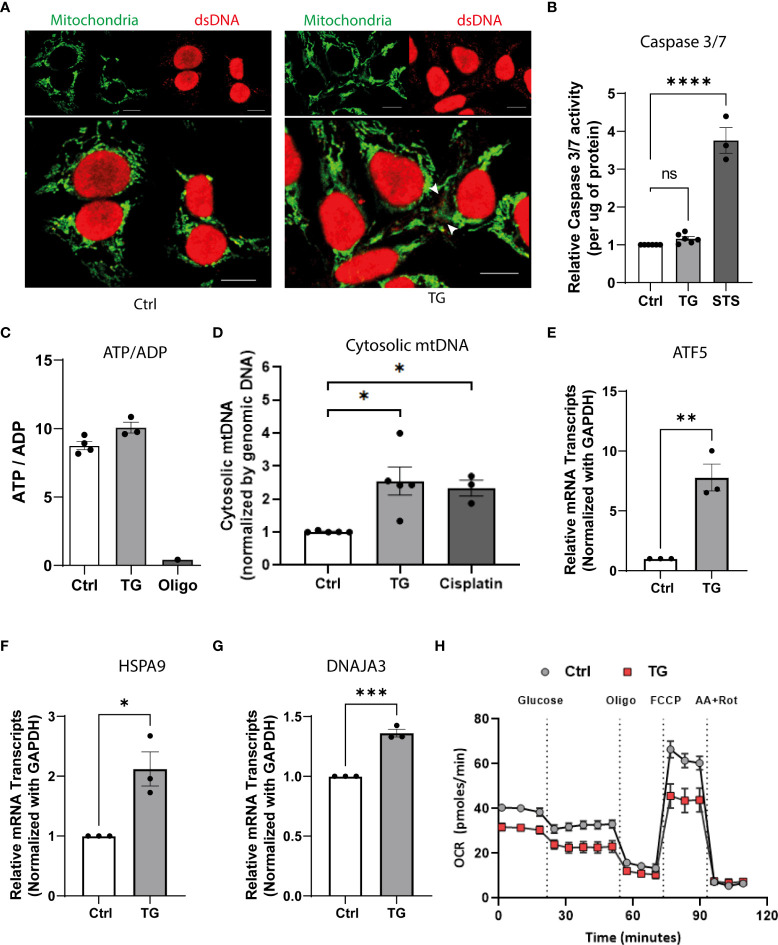
ER stress induces mitochondrial damage leading to mtDNA leakage to the cytosol. **(A)** Confocal imaging of EndoC-βH1 cells exposed to TG (0.1µM; 24h) and stained with anti-dsDNA (red) and anti- mitochondria (green); scale = 10µm. **(B)** Relative caspase 3/7 activity (expressed per µg of protein) in EndoC-βH1 cells exposed to TG (0.1µM; 24h) or Staurosporine (1µM, 2h). **(C)** the ATP-on-ADP ratio was calculated after determination of intracellular ATP and ADP levels by HPLC in EndoC-βH1 cell after exposure to TG (0.1 µM, 24h) or oligomycin (1 µM, 24h); ATP/ADP ratios are plotted as a mean ± SEM (n=3). **(D)** mtDNA cytosolic expression in EndoC-βH1 cell exposed to TG (0.1 µM, 24h) or Cisplatin (1µM, 24h). The results are normalized with nuclear DNA and presented as a mean ± SEM (n=5). **(E–G)** Relative mRNA expression of mitochondrial stress genes **(E)** ATF5, **(F)** HSPA9 and, **(G)** DNAJA3 in EndoC-βH1 cells exposed to TG. **(H)** Analysis of metabolic function by measuring oxygen consumption rate (OCR; pmoles/min) in a Seahorse XF analyzer. The graph represents the mean ± SEM (n=5) statistically analyzed by student’s t-test. Significance is indicated by *P < 0.05, **P < 0.01, ***P < 0.001 and ****P < 0.0001.

To characterize the source of nucleic acids and discriminate free cytosolic dsDNA from mitochondria dsDNA, we combined dsDNA staining with mitochondria labelling. Immunohistochemistry analyses presented in **Figure 4A** demonstrate the presence of free cytosolic dsDNA outside mitochondria in TG exposed EndoC-βH1 cells (**Figure 4A**). The absence of apoptosis, as assessed by caspase 3/7 activity (**Figure 4B**), and changes in cellular ADP/ATP ratio (**Figure 4C**) after TG treatment suggests that dsDNA does not originate from damaged nuclei of pro-apoptotic cells but rather from mitochondrial leakage. To validate the mitochondrial origin of the cytosolic dsDNA, we used a PCR-based approach combined with primers directed against mtDNA sequences in order to quantify mtDNA leakage. As depicted in **Figure 4D**, TG treatment led to increase expression of mtDNA in the cytosol of EndoC-βH1 cells whereas total mtDNA content remained unchanged (**Figure S3**). Of note, the presence of cytosolic mtDNA also correlated with mitochondria stress, as shown by increased expression of Activating Transcription Factor 5 (ATF5), Heat Shock Protein Family A (Hsp70) Member 9 (HSPA9) and DnaJ homolog subfamily A member 3 (DNAJA3) (**Figures 4E–G**), and decrease in mitochondrial oxygen consumption rate (OCR), as assessed by Seahorse flux analyzer in both basal and uncoupled state (**Figure 4H**).

### ER stress induction triggers IL8-dependent neutrophil chemotaxis

**Figure 5:**
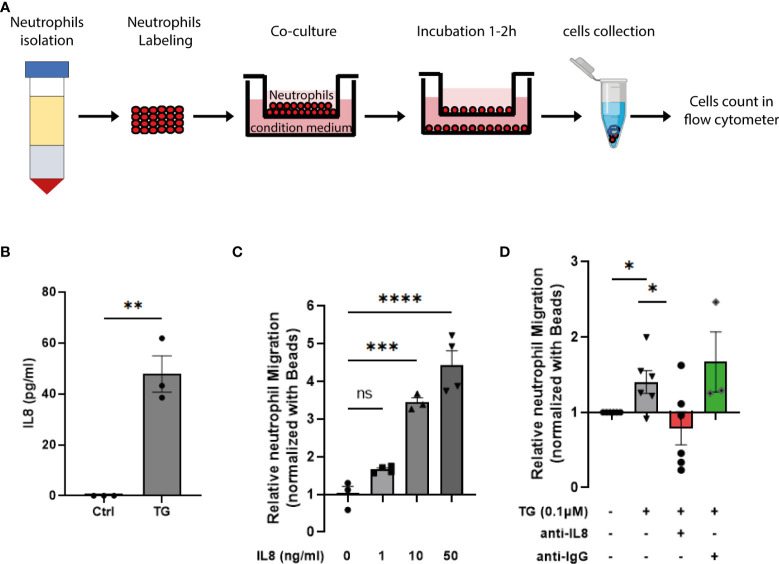
ER stress induction triggers IL8-dependent neutrophil chemotaxis. **(A)** Schematic representation of the transwell migration assay. **(B)** human IL8 ELISA performed on EndoC-βH1 cell supernatant in presence or absence of TG (0,1µM for 24h) **(C)** Relative neutrophil chemotaxis to recombinant human IL8 (1 – 50 ng/ml) **(D)** Relative neutrophil migration to conditioned medium from EndoC-βH1 treated cells. The conditioned medium from TG exposed cells were pre-incubated with either anti-IL8 blocking antibody (250 ng/ml) or anti-IgG (250 ng/ml)as negative control for 10 min. Date are expressed as mean ± SEM (n=3-5) of relative migration to the untreated conditioned medium and statistically analyzed by student’s t-test. Significance is indicated by *P < 0.05, **P < 0.01, ***P < 0.001, ****P < 0.0001, and ns, not significant.

In order to determine the consequence of cellular stress and the induced type-I interferon response in the beta-cell crosstalk with innate immune cells, we first assessed the impact of ER stress on cytokine production by primary human islets. As shown on **Figure S4**, TG exposure triggered the secretion of small amount of IL6 and high level of IL8, one of the most potent neutrophils chemoattractant. To test the neutrophils migration capacity towards stressed beta cells, we designed a trans-well migration assay (**Figure 5A**) and evaluated IL8 secretion by EndoC-βH1 upon TG treatment using IL8 specific ELISA. In these assays, we confirmed the increased IL8 secretion by beta cells in response to TG (**Figure 5B**), the IL8 dose-dependent neutrophils migration (**Figure 5C**) and demonstrated a role for the IL8 secreted upon TG treatment in neutrophils chemotaxis (**Figure 5D**).

## Discussion

Several studies have reinforced a role for IFN-I in the early stages of beta-cell autoimmunity, connecting innate and adaptive immune cells (36–38). While viruses have been proposed as initial cause (39, 40), the recent demonstration of the implication of mtDNA leakage in the development of SLE, beg to reconsider the participation of an endogenous trigger of the IFN-I response in other autoimmune diseases (41). Yet, despite the close link established between the increased MDA5 expression and T1D development, mitochondria dsRNA has been excluded as potential trigger for IFN-I response in beta-cells (42). In the present study, we describe two novel phenomena resulting from ER stress occurring in human beta-cells: i) a cytoplasmic accumulation of mitochondria-derived mtDNA associated with an activation of type I interferon response and ii) an increased IL8 secretion that triggers neutrophil migration. While our results suggest that cellular stress, independently of the inflammatory milieu, can initiate innate immunity *via* release of mtDNA, we cannot rule out that other pathways activated upon the unfolded Protein Response may regulate IL8 expression and secretion (43, 44).

In our experiment, the use of the sarco/endoplasmic reticulum Ca^2+^ ATPase (SERCA) pump blocker TG highlights the impact of dysregulated Ca^2+^ homeostasis in beta-cell function and in the ER-mitochondria crosstalk. In this condition, the mitochondria usually acts as a buffer to dampen the elevation in cytosolic Ca^2+^ level. As a consequence, the possible increased Ca^2+^ level in mitochondrial matrix (45) could underly the observed mitochondrial stress, which is reflected by increased expression of ATF5, HSPA9 and DNAJA3, and reduced OCR. The mtDNA leakage observed in response to ER stress can either be a consequence of altered mitochondrial dynamics, such as enhanced fragmentation, or the result of an active process *via* Ca^2+^-triggered opening of the mitochondrial permeability transition pore (15), both leading to the loss of mitochondrial integrity and release of matrix components. Among them, mtDNA has been proposed as a key driver in the development of type-I interferonopathy (46). As such, the increased cytosolic mtDNA and activation of cytosolic dsDNA sensor cGAS observed in stressed EndoC-βH1 cells echoes recent work on the role of mtDNA in beta-cell senescence after metabolic stress induced by chronic high-fat diet feeding in mice (47).

Of note, while supporting elements in favor of the implication of beta-cell ER stress in T1D pathology have been provided by careful imaging of nPOD sections (48), it remains difficult to draw definitive conclusion on whether ER stress is a cause or rather a consequence of innate immune cell infiltration.

In T1D, the induction of the UPR response to stress has been mostly described as an adaptive response of beta-cell to inflammatory environment. Yet, the recent association between obesity and T1D supporting the “accelerator hypothesis” that the rising demand for insulin during obesity could lead to autoimmunity may position the ER stress as possible cause for immune cell infiltration (49).

In mice, neutrophils were shown to be the first in line, detected in 2 weeks old mice while T1D development classically occurs after 14 weeks (50). In humans, even if their role is less clear, the presence of neutrophils infiltrating the pancreas in presymptomatic T1D subjects (51), combined to the role of NETs in priming DC and T cell activation, suggests that neutrophils orchestrate the induction of the adaptive immune response. The IL8-dependent neutrophil migration measured in our experiments, previously reported in beta-cell line after acidification (52), is in line with these observations and the high serum IL8 level detected in newly diagnose T1D (53).

Altogether, our data provide evidence of the ER stress participation in activating the innate immune response, leading to the recruitment of immune cells as a sign of insulitis triggered during initial stage of T1D. Although the molecular mechanism(s) leading to mtDNA leakage remains unclear and deserve deeper investigation of the ER-mitochondria crosstalk (e.g. MAM junctions, oligomerization of the Ca^2+^ channels and opening of mitochondrial permeability transition pore under cellular stress), the result presented may connect changes in metabolism with the development of autoimmunity and drag toward the identification of new potential targets to keep beta-cells protected from immune attacks.

## Data availability statement

The raw data supporting the conclusions of this article will be made available by the authors, without undue reservation.

## Author contributions

SV designed and performed the experiments and wrote the first draft of the manuscript,. MD, JL, FO and FC performed the experiments. BG and AZ supervised the project and wrote the manuscript. All authors contributed to the article and approved the submitted version.

## Funding

This work is supported by DON Foundation and the Dutch Diabetes Research Foundation, JDRF and by IMI2-JU under grant agreement No 115797 (INNODIA) and No 945268 (INNODIA HARVEST). This Joint Undertaking receives support from the Union’s Horizon 2020 research and innovation program and “EFPIA”, “JDRF” and “The Leona M. and Harry B. Helmsley Charitable Trust”.

## Conflict of interest

The authors declare that the research was conducted in the absence of any commercial or financial relationships that could be construed as a potential conflict of interest.

## Publisher’s note

All claims expressed in this article are solely those of the authors and do not necessarily represent those of their affiliated organizations, or those of the publisher, the editors and the reviewers. Any product that may be evaluated in this article, or claim that may be made by its manufacturer, is not guaranteed or endorsed by the publisher.

## Supplementary material

SUPPLEMENTARY FIGURE S1
**(A)** cGAS protein expression in EndoC-βH1 after exposure to TG (0,1µM for 24h). Expression of β-actin is used as loading control. **(B)** densitometric quantification of cGAS expression. Data are shown as average of 3 independent experiments and as relative intensity using β-actin as reference.

SUPPLEMENTARY Figure S2
**(A)** MDA5 gene expression after MDA5 specific siRNA transfection or scrambled siRNA transfection. **(B)** IFNβ gene expression after MDA5 specific siRNA transfection or scrambled siRNA transfection. **(C)** cGAS gene expression after cGAS specific shRNA transduction or control shRNA lentivirus transduction. **(D)** IFNβ gene expression gene expression after cGAS specific shRNA transduction or control shRNA lentivirus transduction.

SUPPLEMENTARY Figure S3 Total mtDNA amount determined by qPCR in EndoC-βH1 cells after TG exposure.

SUPPLEMENTARY Figure S4 Cytokine profile expression determined by Luminex assay on primary islet supernatant after TG exposure (n=1 pancreas donor. The results are representative of 3 independent measurement).
