# Supplementary figures and images for: ER stress promotes mitochondrial DNA mediated type-1 interferon response in beta-cells and interleukin-8 driven neutrophil chemotaxis
Source: Front Endocrinol (Lausanne). 2022 Sep 12;13:991632. doi: 10.3389/fendo.2022.991632 (PMC9511040; doi:10.3389/fendo.2022.991632)

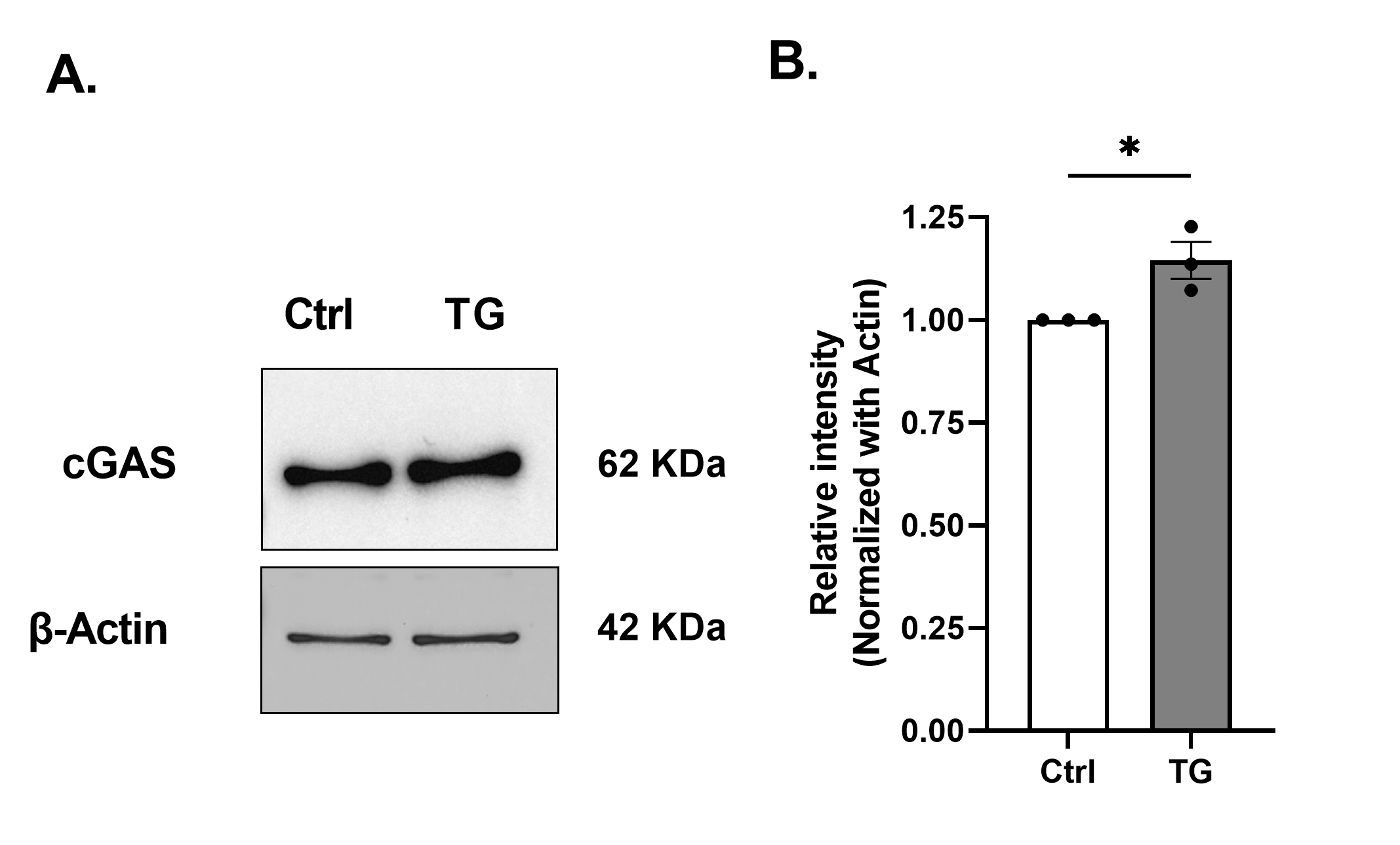

Supplement: SUPPLEMENTARY FIGURE S1 — (A) cGAS protein expression in EndoC-βH1 after exposure to TG (0,1µM for 24h). Expression of β-actin is used as loading control. (B) densitometric quantification of cGAS expression. Data are shown as average of 3 independent experiments and as relative intensity using β-actin as reference. [file Image_1.tif]

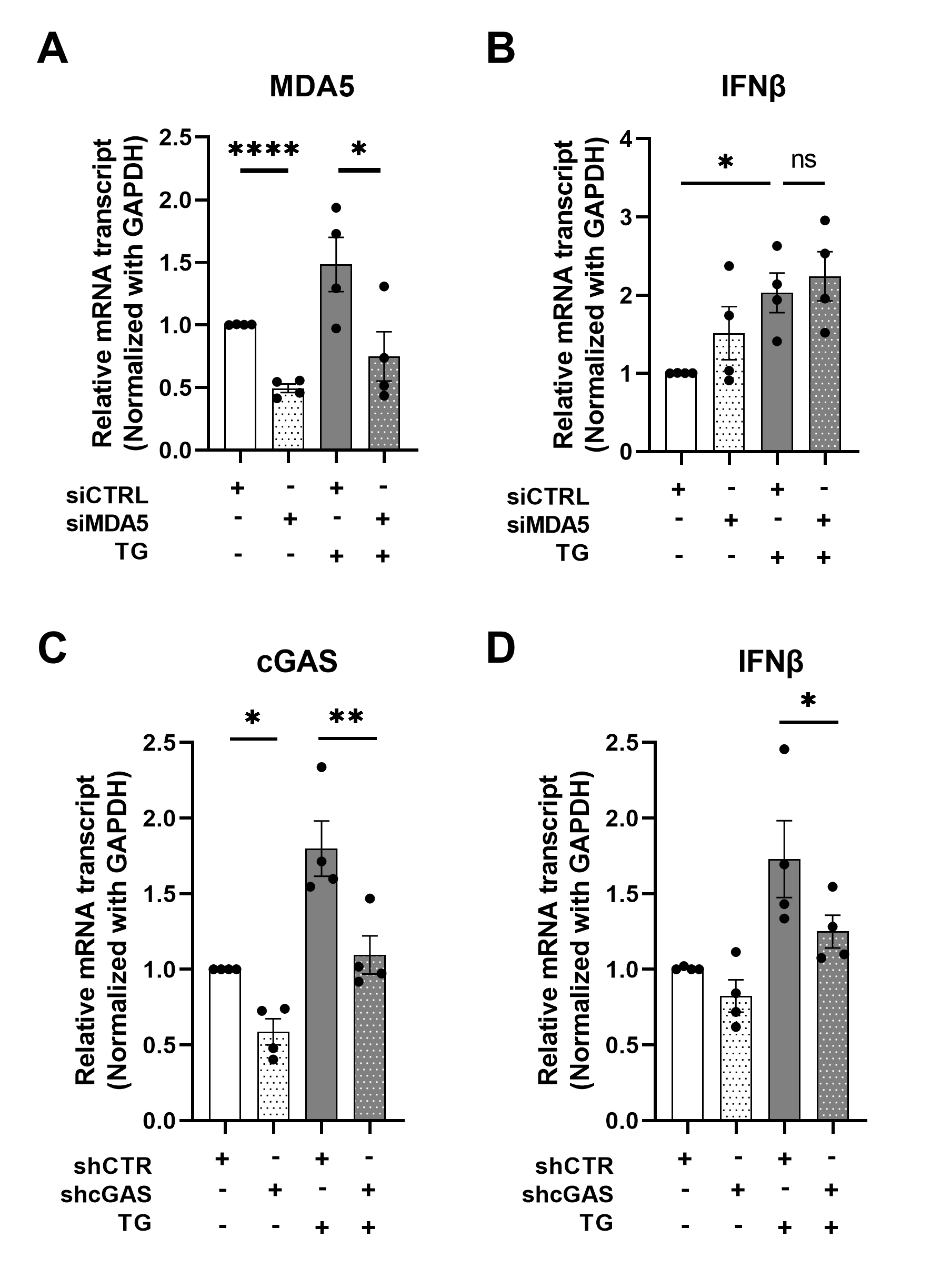

Supplement: SUPPLEMENTARY Figure S2 — (A) MDA5 gene expression after MDA5 specific siRNA transfection or scrambled siRNA transfection. (B) IFNβ gene expression after MDA5 specific siRNA transfection or scrambled siRNA transfection. (C) cGAS gene expression after cGAS specific shRNA transduction or control shRNA lentivirus transduction. (D) IFNβ gene expression gene expression after cGAS specific shRNA transduction or control shRNA lentivirus transduction. [file Image_2.tif]

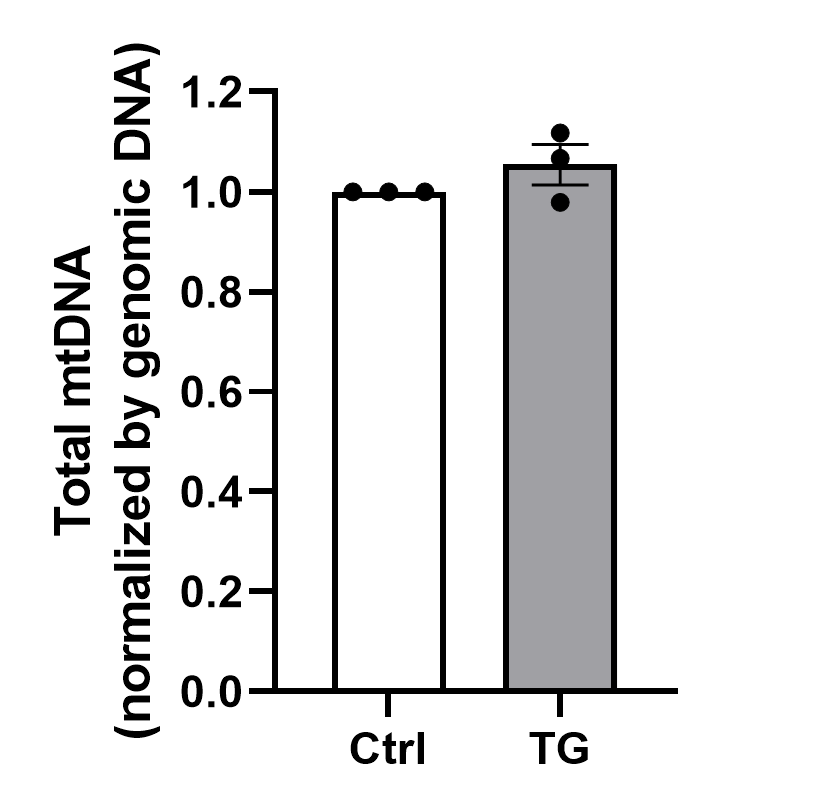

Supplement: SUPPLEMENTARY Figure S3 — Total mtDNA amount determined by qPCR in EndoC-βH1 cells after TG exposure. [file Image_3.tif]

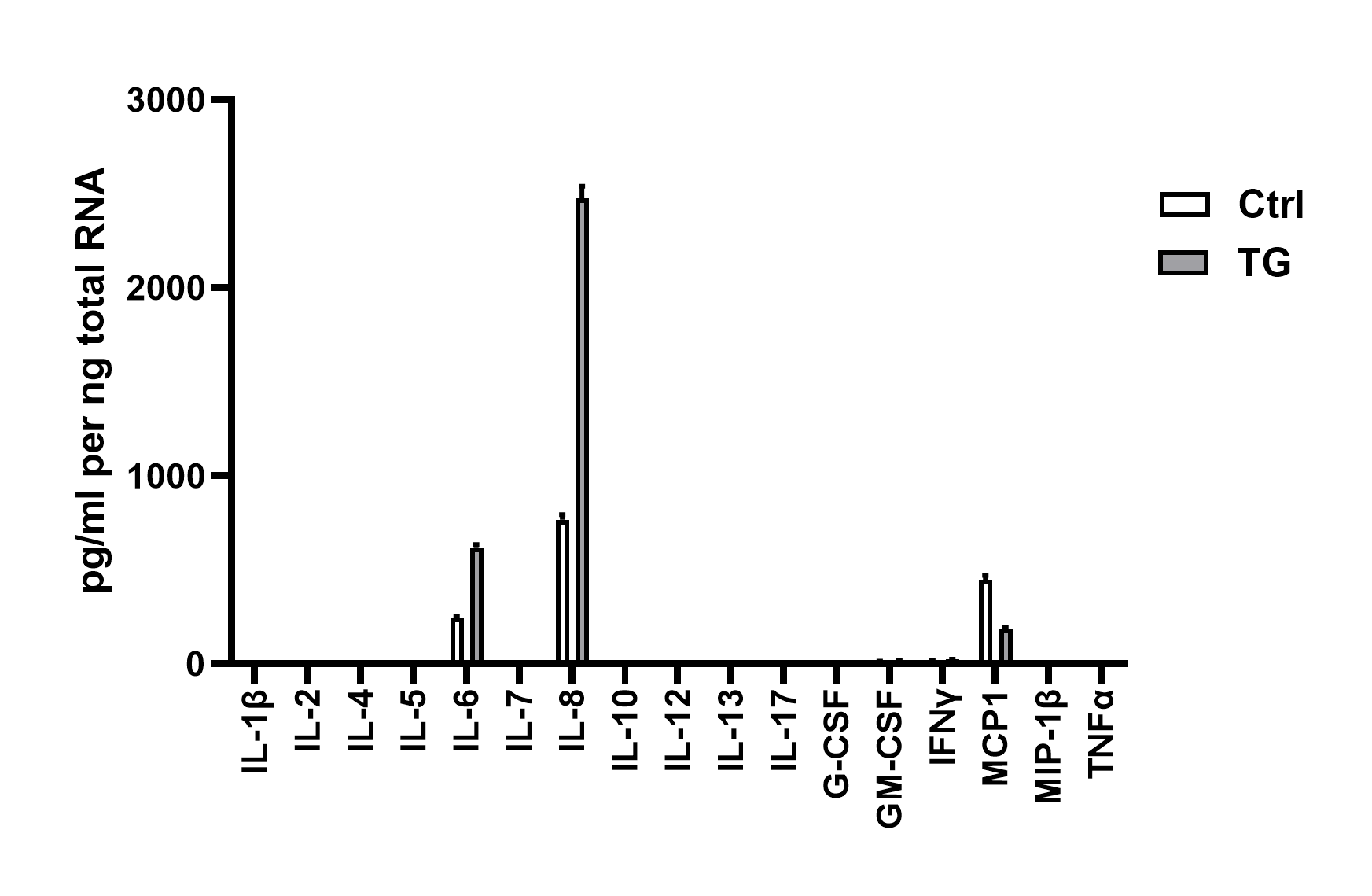

Supplement: SUPPLEMENTARY Figure S4 — Cytokine profile expression determined by Luminex assay on primary islet supernatant after TG exposure (n=1 pancreas donor. The results are representative of 3 independent measurement). [file Image_4.tif]
